# A model for presenting accelerometer paradata in large studies: ISCOLE

**DOI:** 10.1186/s12966-015-0213-5

**Published:** 2015-04-20

**Authors:** Catrine Tudor-Locke, Emily F Mire, Kara N Dentro, Tiago V Barreira, John M Schuna, Pei Zhao, Mark S Tremblay, Martyn Standage, Olga L Sarmiento, Vincent Onywera, Tim Olds, Victor Matsudo, José Maia, Carol Maher, Estelle V Lambert, Anura Kurpad, Rebecca Kuriyan, Gang Hu, Mikael Fogelholm, Jean-Philippe Chaput, Timothy S Church, Peter T Katzmarzyk, for the ISCOLE Research Group

**Affiliations:** Pennington Biomedical Research Center, 6400 Perkins Road, Baton Rouge, LA 70808 USA; Syracuse University, Syracuse, USA; Oregon State University, Corvallis, USA; Tianjin Women’s and Children’s Health Center, Tianjin, China; Children’s Hospital of Eastern Ontario Research Institute, Ottawa, Canada; University of Bath, Bath, UK; School of Medicine Universidad de los Andes, Bogota, Colombia; Kenyatta University, Nairobi, Kenya; University of South Australia, Adelaide, Australia; Centro de Estudos do Laboratório de Aptidão Física de São Caetano do Sul (CELAFISCS), Sao Paulo, Brazil; Faculdade de Desporto, University of Porto, Porto, Portugal; University of Cape Town, Cape Town, South Africa; St. Johns Research Institute, Bangalore, India; University of Helsinki, Helsinki, Finland

**Keywords:** Exercise, Motor activity, Methods, Data collection, Evaluation studies

## Abstract

**Background:**

We present a model for reporting accelerometer paradata (process-related data produced from survey administration) collected in the International Study of Childhood Obesity Lifestyle and the Environment (ISCOLE), a multi-national investigation of >7000 children (averaging 10.5 years of age) sampled from 12 different developed and developing countries and five continents.

**Methods:**

ISCOLE employed a 24-hr waist worn 7-day protocol using the ActiGraph GT3X+. Checklists, flow charts, and systematic data queries documented accelerometer paradata from enrollment to data collection and treatment. Paradata included counts of consented and eligible participants, accelerometers distributed for initial and additional monitoring (site specific decisions in the face of initial monitoring failure), inadequate data (e.g., lost/malfunction, insufficient wear time), and averages for waking wear time, valid days of data, participants with valid data (≥4 valid days of data, including 1 weekend day), and minutes with implausibly high values (≥20,000 activity counts/min).

**Results:**

Of 7806 consented participants, 7372 were deemed eligible to participate, 7314 accelerometers were distributed for initial monitoring and another 106 for additional monitoring. 414 accelerometer data files were inadequate (primarily due to insufficient wear time). Only 29 accelerometers were lost during the implementation of ISCOLE worldwide. The final locked data file consisted of 6553 participant files (90.0% relative to number of participants who completed monitoring) with valid waking wear time, averaging 6.5 valid days and 888.4 minutes/day (14.8 hours). We documented 4762 minutes with implausibly high activity count values from 695 unique participants (9.4% of eligible participants and <0.01% of all minutes).

**Conclusions:**

Detailed accelerometer paradata is useful for standardizing communication, facilitating study management, improving the representative qualities of surveys, tracking study endpoint attainment, comparing studies, and ultimately anticipating and controlling costs.

**Trial registration:**

ClinicalTrials.gov: NCT01722500

**Electronic supplementary material:**

The online version of this article (doi:10.1186/s12966-015-0213-5) contains supplementary material, which is available to authorized users.

## Background

Paradata are process-related data produced as a result of survey administration [1]. Paradata can be used to facilitate study management, improve the representative qualities of surveys, track study endpoint attainment, and ultimately anticipate and control costs [1-3]. Survey data have evolved beyond only self-reported metrics, and now large scale health surveys also include objective monitoring of physical activity [4,5], sedentary behaviors [6], and/or sleep episode time [7] using body-worn accelerometer-type devices. Accelerometer paradata are thus administrative data related to accelerometer data collection, management, and treatment. Sources of such paradata include the electronic devices and accelerometer files themselves, but also checklists, flow charts [8], and other forms of process-related data collection used to streamline each of these administrative stages of survey-based research.

Accelerometer paradata are inconsistently reported [8], and are typically limited only to reports of the number of valid days of data collected and average wear time [9]. However, these data can also include, but are not limited to, the number of accelerometers distributed relative to the targeted population, the number of lost/malfunctioning accelerometers, and the number of cases culled at each study stage including during participant enrollment and data collection and processing, leading to the finalized locked data set. The purpose of this novel report is to present the accelerometer paradata collected in the International Study of Childhood Obesity Lifestyle and the Environment (ISCOLE), a multi-national investigation of over 7000 children (averaging 10.5 years of age) sampled from 12 different developed and developing countries across five continents. We provide lessons learned and recommendations to systematically describe the capture and reporting of these paradata for future design, budgeting, harmonization, and comparability of accelerometry-based studies.

## Methods

### The ISCOLE study

ISCOLE was a cross-sectional study of lifestyle and environmental factors that may influence children’s obesity. The overarching ISCOLE protocol was approved by Pennington Biomedical Research Center’s Institutional Review Board (IRB). In addition, each ISCOLE study site received protocol approval from their institutional ethics committee. The design and overall methods of the ISCOLE study have been previously published [10] and the detailed accelerometry Manual of Procedures for the collection, management, and treatment of accelerometer data published in an electronic supplementary file to a separate ISCOLE article [11]. Briefly, a 24-hr/day, 7-day waist-worn accelerometer (GT3X+, ActiGraph, LLC, Pensacola, FL, USA) data collection protocol (removal only for water-based activities) was implemented with over 500 children from each of the participating sites around the world: Australia, Brazil, Canada, China, Colombia, Finland, India, Kenya, Portugal, South Africa, the United Kingdom (U.K.), and the United States of America (U.S.). Sites targeted one or two grades (e.g., grade 4 in the U.S. site, grades 4 and 5 in the Colombia site) within multiple local schools that would most likely identify the greatest number of 10 year old children, the specific target for the ISCOLE study. Accelerometers were distributed in schools. In order to facilitate data collection and participant adherence amongst peers from the same classrooms, children outside the age range were allowed to participate but their data were later deemed ineligible. Children were asked to wear accelerometers for 7 consecutive days (not including the initial familiarization period of the first day and the morning of the final day before accelerometer retrieval) to capture their free-living behavior independent from other ISCOLE testing requirements. However, local sites determined other logistics of data collection on a school-by-school basis, including whether accelerometers were distributed subsequent to anthropometric assessment and whether other data were collected on the same day, or on a different day [10]. Additional protocol details are presented in the ISCOLE accelerometry Manual of Procedures [11].

Following the monitoring period, accelerometers were retrieved from children at their schools and accelerometer data were identified as adequate or inadequate before uploading to the secure U.S.-based ISCOLE Data Management Website housed and managed at Wake Forest University (Winston-Salem, NC, U.S.). Inadequate data could result from accelerometer loss or malfunctioning, a refusal to wear, insufficient wear time (i.e., < 4 days or missing one weekend day, each with ≥ 10 hours of waking wear time) determined using ActiLife Version 5.6 software (or higher, as new releases were provided), or other unspecified reasons. If the data were deemed inadequate during this initial review, local sites had the option of conducting a second week of additional monitoring; this decision was made on a case-by-case basis. Sites retained all original raw files as a backup strategy. However, these raw files were also sent to the Coordinating Center (located at the Pennington Biomedical Research Center in Baton Rouge, U.S.) once the local site completed data collection. Quality control checks (including another data query for valid data defined by wear time, number and types of days; details below) and cleaning processes were systematically performed prior to creating a locked data set containing a number of derived physical activity-, sedentary behavior-, and sleep-related variables based on previously published algorithms and definitions [7,12-20]. All acquired data were saved however, and can be manipulated and used in different ways for future analyses as desired.

### Sources of accelerometer paradata

#### Participant checklist (PACK)

Staff at each site were trained to use a common PArticipant ChecKlist (PACK) that was developed to administratively track individual participants’ progress through the ISCOLE study, including accelerometry data collection. The PACK served to record participant identification (PID) numbers, accelerometer serial numbers, distribution and retrieval dates, whether or not data were present and adequate upon retrieval, and whether or not the child was asked to wear an accelerometer for a second week of additional monitoring (and again, all relevant accelerometer-related information asked for the initial monitoring). All data collected on paper forms of the PACK were electronically entered by local study staff into the ISCOLE study coordinating center's secure website. Electronic warnings were automatically issued in the event of missing or implausible entries, and local study staff members were required to resolve all warnings prior to the end of data collection at the site. An automatic query requested reasons why data were inadequate (if this response was selected during the data entry process). No adverse events (e.g., skin irritation) were reported through this prompted and open-ended query system. To increase the reliability of the recorded explanation of data inadequacy, we recommend that future versions of the PACK include such a field, as well as reasons why accelerometers may not have been distributed as expected, and accelerometer initialized start and download dates and times (in addition to distribution and retrieval dates).

#### Accelerometer files

Reportable paradata extracted from all compressed (.ZIP) 1 second .AGD accelerometer files and/or extracted from the processed data (aggregated into 60 second epochs) include 24-hr wear time, waking wear time, a count of valid days (≥10 hours of waking wear time) and valid nights (≥160 minutes of total sleep episode time [7]) of data, a count of participants with valid waking wear time (≥4 valid days of data, including 1 weekend day) and total sleep episode time (≥3 valid nights of data, including one weekend night), and a count of implausibly high values (≥20,000 activity counts/min [21]). Additional definitions and details are presented in (See Additional file 1: Table S1).

As per the monitoring protocol’s design (see the ISCOLE accelerometry Manual of Procedures for details including rationale [11]), a maximum of 7 days from each accelerometer file were screened for possible inclusion in the summary datasets. If the first day of data for any accelerometer files indicated an initialization time other than midnight (standardized during initialization process) or that the device was initialized prior to being placed on the child, then all data collected prior to the first recorded midnight were deleted. In addition, the last day of data was also deleted and any data collected past the scheduled monitoring period in the case of delayed retrieval. Since the monitoring protocol required 24-hour wear, it is also important to emphasize that total sleep episode time [7] was first identified from within the accelerometer data prior to further evaluating it for evidence of non-wear (for details refer to the ISCOLE accelerometry Manual of Procedures [11]) and ultimately producing the final list of derived variables. Specifically, after accounting for total sleep episode time and non-wear time, valid days were considered those with ≥ 10 hours of waking wear time and the locked data set included only those participants with ≥4 valid days of data [4], including 1 weekend day. As described in the ISCOLE accelerometer Manual of Procedures [11], the requirement for at least 1 weekend day was based on known differences between weekdays and weekend days in children [22-24] and also because it has been a common analytical choice as previously applied to NHANES children’s accelerometer data [9].

### Data analysis

**Figure 1:**
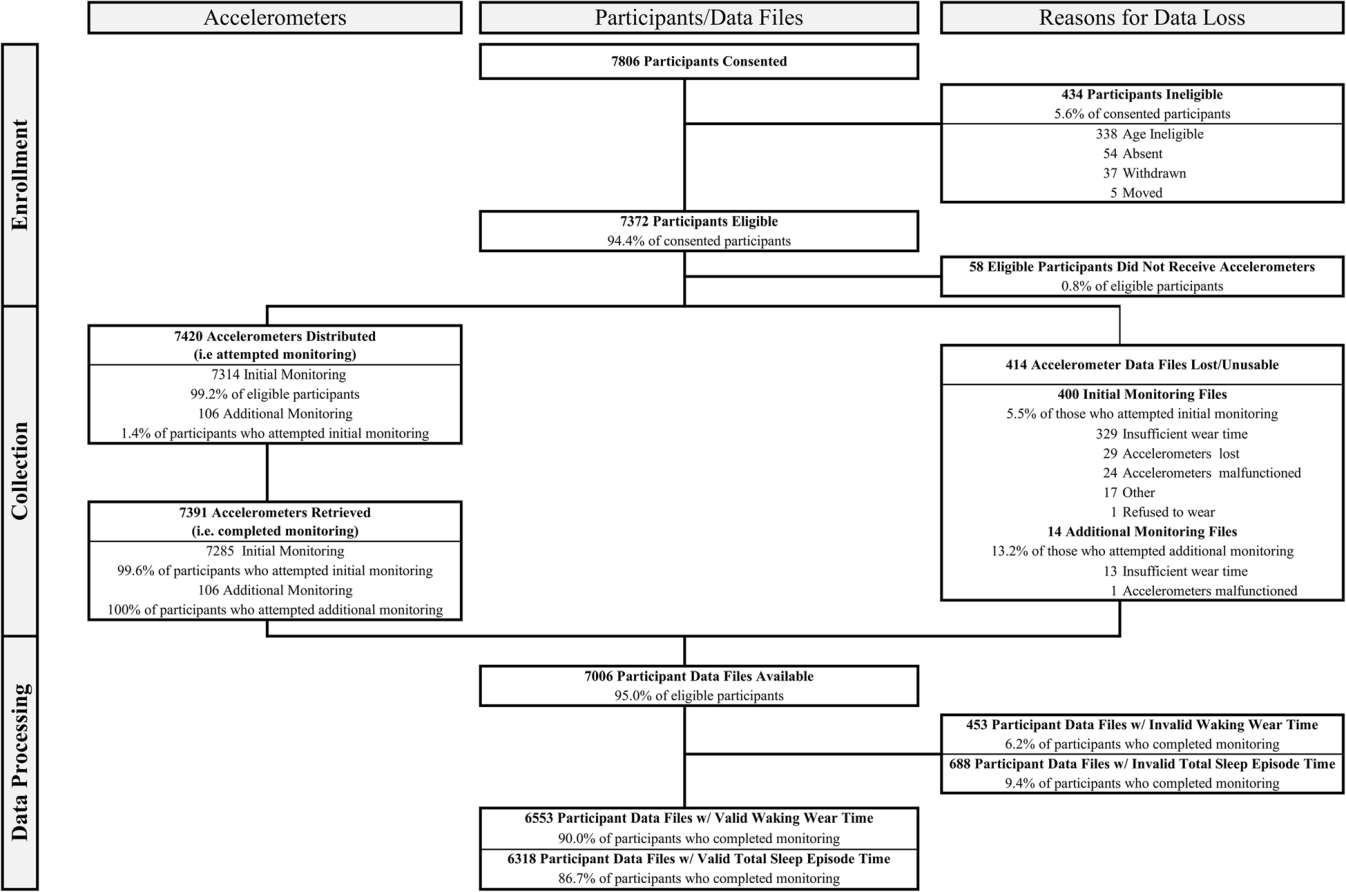
Participant flow chart reflecting the separate study stages of participant enrollment, data collection, and data processing cross-tabulated with trackable data derived from accelerometers, participants/data files, and reasons for data loss at each stage.

Descriptive data (counts, means, standard deviations, ranges, and percentages where appropriate) for each paradata variable defined in (Additional file 1: Table S1) were computed for the entire ISCOLE sample and averaged over sites. We designed the flow chart (Figure 1) to reflect the separate study stages of participant enrollment, data collection, and data processing while highlighting the trackable data derived from accelerometers, participants/data files, and reasons for data loss at each stage.

Once a parent/legal guardian provided informed consent and the individual child provided assent for participation (only when required by local ethics committees), that child was enrolled in ISCOLE and assigned a PID number. A count of unique study PID numbers assigned to enrolled children provided an accurate representation of consented participants. Eligible children were defined during data processing as those aged 9–11 years who did not withdraw or were not dropped from the study for any reason (e.g., moved or absent when anthropometric and/or other non-accelerometry data were being collected). Additional paradata variables in Figure 1 and/or (Additional file 1: Table S1) include reasons for inadequate accelerometer data, the number of participants completing initial additional monitoring (i.e., the number from whom accelerometers were retrieved), the number of files deemed adequate, the number of files culled during data processing, the number with valid waking wear time, the number with valid total sleep episode time, and the number available in the locked data set for analyses. Denominators for calculated percentages are indicated in (Additional file 1: Table S1).

## Results

During the enrollment phase of the study, 7806 participants from the 12 ISCOLE study sites around the world consented to wearing an accelerometer and 7372 (94.4% of consented participants) were deemed eligible to participate. The most frequently reported reason for participant ineligibility was being outside the designated 9–11 year old age range, reflecting our planned recruitment strategy that targeted grades within schools (not age specifically). During the data collection phase, 7314 accelerometers (99.2% of eligible participants) were distributed for initial monitoring. Another 106 accelerometers were re-distributed for additional monitoring (1.4% of those who attempted initial monitoring participated in additional monitoring and 26.5% of those who were eligible for additional monitoring participated in additional monitoring).

Also during this phase, 414 accelerometer data files (across both initial and additional monitoring opportunities) were deemed inadequate. The most common cause of this loss was due to insufficient wear time (assessed locally at each site using ActiLife software). Across all sites, 926 unique accelerometers were used, representing an assessment capacity of approximately 8 participants per accelerometer. Only 29 accelerometers were lost during the implementation of ISCOLE worldwide. This represents a 3.1% loss rate relative to the number of unique accelerometers used and a 0.4% loss rate relative to the total number of accelerometers distributed over the course of both initial and additional monitoring frames conducted across the 12 study sites. The data processing phase considered 7006 unique participant files (95% of eligible participants). The primary reason for data loss after this point was not meeting the ISCOLE study requirements for wear time and number and types of days and/or nights after accounting for total sleep episode time. The final locked data file consisted of 6553 participants files (90.0% relative to participants who completed monitoring) with valid waking wear time and 6318 participants (86.7% relative to participants who completed monitoring) with valid total sleep episode time. Relative to the denominator of eligible participants the congruent percentages were 88.9% and 85.7%.

Participants in the locked data set averaged 6.5 valid days of data out of a possible 7 days as per protocol, (92.9%), and 1366.8 minutes/day (22.8 hours/day) of wear time over the 24-hour period (95% of 1440 hours/day as per protocol). Of these, an average of 888.4 minutes/day (14.8 hours) was considered to be waking wear time. ISCOLE sites reported 25 cases of accelerometer malfunctioning (considering both initial and additional monitoring) and we documented 4762 minutes with implausibly high activity count values (≥20,000 activity counts/minute [21,25]) from 695 unique ISCOLE participants (9.4% of eligible participants and <0.01% of all minutes). These high activity count values were considered indicative of accelerometer malfunction [21,25] and therefore we re-set associated minutes to non-wear or sleep [7] depending on the classification of the surrounding minutes.

## Discussion

### A paradata reporting model

Successful implementation of ISCOLE at 12 international study sites demanded concerted attention to planning and logistics. A product of this ambitious process was our ability to capture and report informative survey paradata, especially those focused on the administration of accelerometry-based measures of physical activity, sedentary behavior, and sleep-related behaviors. Others [8] have suggested that checklists and flow charts can facilitate study administration. However, we are unaware of any other studies of this scope that have published comparably detailed accelerometer paradata. The European Youth Health Study (EYHS) reported analyses on available “valid and complete” data collected from 9–10 year olds [26]. The European HELENA study [27] reported that “88% of the adolescents had 4 or more valid days” and mean wear time for this subgroup was 12.9 ± 1.5 hours/day. No other accelerometer paradata were reported. In ISCOLE, we combined the use of checklists and flowcharts with direct data queries of accelerometer files to develop a much more detailed reporting model. Such detailed reporting of accelerometer paradata is useful for standardizing communication, facilitating study management, improving the representative qualities of surveys, tracking study endpoint attainment, assuring quality control, comparing studies, and ultimately anticipating and controlling costs.

### Examples of potential Use

The utility of these paradata can be illustrated with a few examples. The ISCOLE recruiting strategy intentionally targeted grades within schools that would most likely yield the participants’ target age range of 9–11 years. We had advance knowledge that at least some children in these grades would be outside of this age range; however, we decided that all children in solicited classrooms would be invited to participate in an effort to enhance adherence and avoid any sense of inclusionary versus exclusionary social dynamics. As a result, accelerometer data from a small percentage (338/7806 = 4.3%) of consented participants outside the intended age range were dropped from further analyses. Capture and presentation of this specific paradata variable helps us judge that this loss was reasonable and ultimately sets a benchmark for future study planning.

We also allowed study sites the flexibility to conduct additional monitoring if initial monitoring failed to provide adequate data. This decision was made on a case-by-case basis at the discretion of local study site staff. Relatively few participants (106 across all sites or 1.4% of those who attempted initial monitoring) participated in additional monitoring. We are aware that the choice to offer additional monitoring was affected by many and varied factors, including logistical concerns of tending to solitary participants at a completed school while moving on to fuller implementation at a subsequently scheduled school. All accelerometers were retrieved from participants who attempted additional monitoring (i.e., 100% completed additional monitoring), and only 13.2% provided inadequate data. Therefore, we believe that the additional effort was worthwhile for the majority of respondents who agreed to this additional monitoring. These paradata can help researchers determine the return on the effort of conducting additional monitoring, gauge its value relative to their research questions and resources, and plan for its potential yield accordingly.

As a final example of the usefulness of accelerometer paradata, local sites used ActiLife software to determine whether there was sufficient wear time detected and record their findings using the PACK prior to uploading to the ISCOLE data management website. Although inadequate data could result from accelerometer loss or malfunctioning, a refusal to wear, or other unspecified reasons, the predominant concern during both initial and additional monitoring, was insufficient wear time as determined from the ActiLife software. In the process of finalizing our locked data set, we first removed total sleep episode time before employing an algorithm to determine waking wear time. As a result of this two-step process, we identified and removed an additional 6.2% of participant files (of those completing initial or additional monitoring) that did not meet ISCOLE analysis requirements for valid waking wear time. The ActiLife software served as a useful tool to quickly make on-site determinations of wear time sufficiency and inform staff whether or not additional monitoring should be attempted; however, these paradata indicate that accounting for total sleep episode time in a 24-hour wear time protocol will further limit the number of valid participants in the locked data set.

### Establishing benchmarks

ISCOLE issued and re-issued 926 unique accelerometers 7420 times (considering both initial and additional monitoring) to 7314 eligible participants across 12 study sites to generate the locked data set of 6553 participant files with valid waking wear time. Only 29 accelerometers were lost, and there were only 25 reported cases of accelerometer malfunctioning. Further, we identified 4762 minutes with implausibly high activity count values across 695 unique participants. However, it is difficult to interpret these accelerometer loss/performance findings as mentioned above since we know of no other comparable paradata at this time despite recommendations for such reports [8]. Similar future reports will help evaluate the magnitude and meaningfulness of these potential problems, and reporting it here will provide essential comparative data.

ISCOLE implemented a 7-day 24-hour wearing requirement protocol and collected data in groups of similarly aged children by distributing accelerometers through schools. Within the locked dataset that included data from 12 international study sites, participants averaged 22.8 hours of wear (of which 14.8 hours were considered waking wear time) over 6.5 valid days of data. In addition, participants provided 8.8 hours of total sleep episode time over 5.5 valid nights of data that can be scrutinized in subsequent analyses for study of sleep-related variables. We know of no other accelerometer study that has reported accelerometer paradata in a similarly detailed manner at this time. Future reports will illuminate the influence of different protocol choices on accelerometer paradata variables.

## Conclusions

Capture and reporting of accelerometer paradata beyond simple wear time statistics requires systematic data collection. We recommend that frontline data collection staff be trained to use a study document (e.g., the PACK described above) specifically designed to track participant enrollment and accelerometer data collection. As a legitimate source of study data, this checklist-type document is more than merely an administrative tool, and should receive a similar level of data quality assurance attention. Building on the original flow chart suggested by Matthews et al. [8], we offer a more detailed version in Figure 1 that organizes the data flow into the study phases of enrollment, data collection, and data process, ultimately leading to the locked data set. (Additional file 1: Table S1) presents additional detail on the multiple suggested accelerometer paradata variables, customized to the ISCOLE study design. Other researchers may use this as an editable foundation for organizing and reporting their own accelerometer paradata. Many of the values in (Additional file 1: Table S1) will serve as preliminary benchmarks to facilitate comparisons between studies. As more accelerometer paradata are reported, a clearer and more harmonic picture of this important administrative information will emerge and accelerate understanding and implementation of objective monitoring of physical activity, sedentary behavior, and sleep-related behavior in free-living populations.

## Additional file

Additional file 1:
**Paradata variables, definitions, and descriptive data from ISCOLE study sites.**
