# Supplementary material for: A model for presenting accelerometer paradata in large studies: ISCOLE
Source: Int J Behav Nutr Phys Act. 2015 Apr 20;12:52. doi: 10.1186/s12966-015-0213-5 (PMC4409714; doi:10.1186/s12966-015-0213-5)
Supplement: Additional file 1: — Paradata variables, definitions, and descriptive data from ISCOLE study sites. [file 12966_2015_213_MOESM1_ESM.pdf]

Paradata variable	Definition	Total from all sites<br>% if applicable (denominator indicated)	Mean ± SD (range) across sites<br>% range if applicable
Participants consented	Unique participant ID numbers representing number of participants enrolled	7806	650.5 ± 131.1 (541-991)
Status	Dropped/withdrawn/ineligible	434<br>5.6% (relative to participants consented)	36.2 ± 26.4 (7 – 78)<br>1.3 – 11.8%
Reasons for dropped/withdrawn/ineligible	Different reasons for ineligibility	338 Age ineligible<br>54 Absent on day of measurement<br>37 Decided not to participate<br>5 Moved	28.2 ± 24.6 (1 – 71)<br>4.5 ± 7.0 (0 – 21)<br>3.1 ± 4.3 (0 – 11)<br>0.4 ± 0.7 (0 – 2)
Eligible participants	9-11 years of age and not otherwise dropped/withdrawn/ineligible	7372<br>94.4% (relative to participants consented)	614.3 ± 119.1 (525 – 919)<br>88.2 – 98.7%
Accelerometers used	Unique accelerometer ID numbers representing number of instruments used during the study	926	77.2 ± 13.6 (69 – 118)
Accelerometer capacity	Mean number of participants assessed per accelerometer	N/A	8.0* (5.6 – 13.6)

Accelerometers distributed	Accelerometers distributed to participants	Initial monitoring: 7314<br>99.2% (relative to eligible participants)<br><br>Additional monitoring: 106<br>1.4% (relative to accelerometers distributed for initial monitoring)	609.5 ± 119.8 (525 – 914)<br>93.2 – 100.0%<br><br>8.8 ± 10.2 (0 – 26)<br>0 – 4.5%
Accelerometers retrieved	Accelerometers retrieved from participants	Initial monitoring: 7285<br>99.6% (relative to accelerometers distributed for initial monitoring)<br><br>Additional monitoring: 106<br>100.0% (relative to accelerometers distributed for additional monitoring)	607.1 ± 119.8 (524 – 914)<br>97.1 – 100.0%<br><br>100.0% at all sites doing any additional monitoring
Accelerometers lost	Accelerometers not returned by participant	29<br><br>0.4% (relative to accelerometers distributed for initial and additional monitoring)<br><br>3.1% (relative to accelerometers used)	2.4 ± 5.3 (0 – 19)<br><br>0 – 2.9%<br><br>0 – 16.1%

Participants with reported adequate data	Participants with reported adequate data (at least 4 days of data with ≥ 10 hours of wear time, including 1 weekend day) as evaluated by ActiLife software	Initial monitoring: 6914<br>94.5% (relative to accelerometers distributed for initial monitoring)<br><br>Additional monitoring: 92<br>86.8% (relative to accelerometers distributed for additional monitoring)	576.2 ± 113.9 (494 – 884)<br>83.2 – 98.6%<br><br>11.5 ± 9.0 (1 – 25)<br>50.0 – 100.0%
Participants with reported inadequate data	Participants with reported inadequate data (< 4 days of data with ≥ 10 hours of wear time and/or lacking a weekend day with ≥ 10 hours of wear time) as evaluated by ActiLife software	Initial monitoring: 400<br>5.5% (relative to accelerometers distributed for initial monitoring)<br><br>Additional monitoring: 14<br>13.2% (relative to accelerometers distributed for additional monitoring)	33.3 ± 28.0 (8 – 109)<br>1.4 – 16.8%<br><br>1.8 ± 2.6 (0 – 8)<br>0 – 50.0%
Reasons for inadequate data	Reported reasons why adequate data is not present	Initial monitoring:<br>329 Insufficient wear time<br>29 Accelerometer lost<br>24 Accelerometer malfunction<br>1 Refused to wear accelerometer<br>17 Other<br><br>Additional monitoring:<br>13 Insufficient wear time<br>1 Accelerometer malfunction	27.4 ± 23.3 (5 – 86)<br>2.4 ± 5.3 (0 – 19)<br>2.0 ± 2.0 (0 – 6)<br>0.1 ± 0.3 (0 – 1)<br>1.4 ± 4.1 (0 – 14)<br><br>1.6 ± 2.3 (0 – 7)<br>0.1 ± 0.4 (0 – 1)
Participants with objectively determined valid waking wear time	After accounting for total sleep episode time and waking wear time, participants with ≥4 days with ≥ 10 hours of waking wear time, including 1 weekend day	6553<br>90.0% (relative to participants who completed monitoring)	546.1 ± 113.5 (468 – 857)<br>75.8 – 94.6%

Valid days	Mean number of days with ≥ 10 hours of waking wear time from participants with valid waking wear time	N/A	6.5 ± 0.7 (5.8 – 6.9)<br>92.9 % (82.6-98.0%) relative to 7 possible days
24-hr wear time	Mean minutes of wear time over 24-hr period per valid day for participants with objectively determined valid waking wear time	N/A	1366.8 ± 106.6 (1315.3 – 1386.5)
Waking wear	Mean minutes of wear time considering only waking hours per valid day for participants with objectively determined valid waking wear time	N/A	888.4 ± 52.6 (844.7 – 910.1)
Participants with objectively determined valid total sleep episode time	After accounting for total sleep episode time, participants with ≥ 3 nights with ≥ 160 mins of total sleep episode time, including one weekend night	6318<br>86.7% (relative to participants who completed monitoring)	526.5 ± 114.8 (425 – 843)<br>76.1 – 92.2%
Valid nights	Mean number of nights with ≥ 160 mins of total sleep episode time from participants with valid total sleep episode time	N/A	5.5 ± 0.8 (4.8 – 5.8)
Total sleep episode time	Mean total minutes from all nocturnal sleep episodes identified by applied algorithm[3]	N/A	529.0 ± 53.7 (496.2 – 569.4)
Implausibly high activity count values	Minutes and unique participants with values ≥ 20,000 activity counts/minute	4762 minutes from 695 unique participants	396.8 ± 505.6 minutes (16 – 1427) from 57.9 ± 45.0 unique participants (12 – 144)

* Calculated value, no SD
